# Identification of putative pathogenic SNPs implied in schizophrenia-associated miRNAs

**DOI:** 10.1186/1471-2105-15-194

**Published:** 2014-06-17

**Authors:** Xiaohan Sun, Junying Zhang

**Affiliations:** 1 School of Computer Science and Technology, Xidian University, Xi’an 710071, P. R. China; 2 College of Mathematics and Information Science, Weinan Normal University, Weinan 714099, P. R. China

## Abstract

**Background:**

Schizophrenia is a severe brain disorder, and SNPs (Single nucleotide polymorphism) in schizophrenia-associated miRNAs are believed to be one of the important reasons for dysregulation which might contribute to the altered expression of genes and ultimately result in the disease. Identification of causal SNPs in associated miRNAs may have certain significance in understanding the mechanism of schizophrenia.

**Results:**

For the above purposes, a method based on detection of free energy change is proposed for identification of causal SNPs in schizophrenia-associated miRNAs. A miRNA is firstly segmented, and free energy change is computed after adding an SNP into a segment. The method discovers successfully 6 out of 32 known SNPs and some artificial SNPs could cause significant change in free energy, and among which, 6 known SNPs are supposed to be responsible for most cases of schizophrenia in population.

**Conclusions:**

The proposed method is not only a convenient way to discover causal SNPs in schizophrenia-associated miRNAs without any biochemical assay or sample comparison between cases and controls, but it also has high resolution for causal SNPs even if the SNPs are not reported for their very rare cases in the population. Moreover, the method can be applied to discover the causal SNPs in miRNAs associated with other diseases.

## Background

Single nucleotide polymorphism (SNP) is a single nucleotide variation that occurs when a single nucleotide, such as an Adenine (A), replaces one of the other three nucleotide letters: Thymine (T), Cytosine (C) or Guanine (G), and it is an important variation for the diversity among individuals, as well as leading to phenotypes, traits, and diseases [1]. SNPs usually occur in regions where natural selection is acting and fixating the allele of the SNP that constitutes the most favorable genetic adaptation [2], and most SNPs (93%) discovered by genome-wide association studies (GWAS) over the last decade that appear to contribute to human disease risk are not located in protein-coding regions [3-5], suggesting that SNPs regulate gene transcription levels rather than alter the protein-coding sequence or protein structure [4]. Therefore, SNPs located in non-coding regions involved in regulation might be closely associated with disease.

MiRNAs (microRNA) are small 20–24 nucleotide (nt) non-coding RNAs that normally negatively regulate messenger RNAs (mRNAs) translation either via mRNA degradation or repression of mRNA translation [5-7]. Animal genomes harbour numerous small, non-coding miRNAs which post-transcriptionally regulate many protein-coding genes to influence the processes ranging from metabolism, development and regulation of nervous and immune systems to the onset of cancer [8]. To date, hundreds of miRNAs have been identified in the human genome, and they play key roles in a broad range of physiologic and pathologic processes [9]. Indeed, the growing understanding of the regulatory properties and pleiotropic effects of miRNAs on molecular and cellular mechanisms, suggests that alterations in the miRNA/mRNA interaction may contribute to phenotypic variation [10]. SNPs in the miRNAs might affect the expression of multiple target genes by disturbing translation or cleavage of the target mRNAs [11], and exhibit more profound and broader biological effects than SNPs in mRNAs [12]. SNPs in miRNAs affect gene regulation mainly in two ways: either impairing miRNA/mRNA interaction or disturbing miRNA biogenesis, both of which finally result in gene dysregulation.

MiRNA/mRNA interaction can be impaired by SNPs in mature miRNAs. SNPs in mature mRNAs could create, destroy, or modify the efficiency of miRNA in binding to 3′-untranslated region (UTR) of target mRNAs [13-15], hence they might cause post-transcriptional dysregulation due to the stringent recognition requirement in binding mature miRNAs to 3′UTRs of target mRNAs in a sequence-specific manner [16,17].

**Figure 1:**
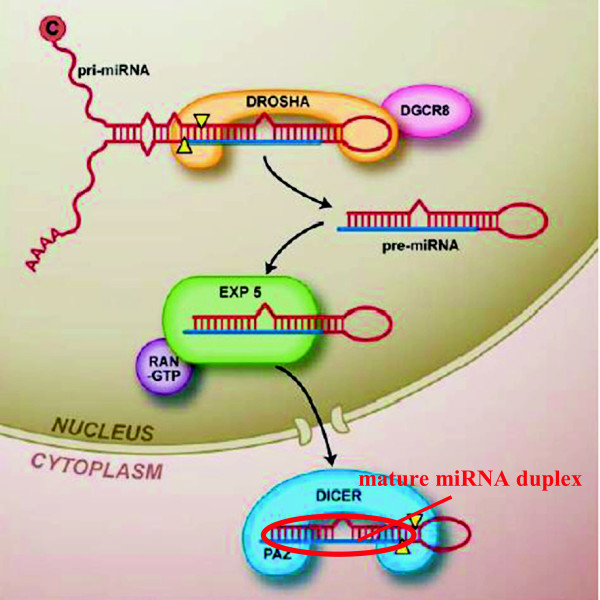
***miRNA biogenesis.*** MiRNAs are processed from hairpin-containing primary transcripts (pri-miRNAs). Pri-miRNA is cleaved firstly by Drosha to produce precursor miRNA (pre-miRNA), and then by Dicer to yield an imperfect miRNA:miRNA* duplex (mature miRNA duplex) about 22 nucleotides in length [20]. Although either strand of the duplex may potentially act as a functional miRNA, only the one strand which is incorporated into the RNA-induced silencing complex (RISC) is termed as mature miRNA.

MiRNA biogenesis can be blocked by SNPs in terminal loops and extension duplexes (covering ~25 nt upstream and downstream from the cleavage sites of Drosha in miRNAs) [18]. Maturation of canonical miRNAs is a two-step cleavage of primary miRNA (pri-miRNA) by Drosha and Dicer [19], following which, pri-miRNA is processed into ~22 nt double-stranded RNA product (Figure 1). Successful cleavage can produce miRNA for miRNA/mRNA interaction, but some SNPs might inhibit cleavage, thus block miRNA biogenesis including miRNA processing, strand loading, and so forth [1,12]. In the process of miRNA maturation, compared to cleavage by Dicer, cleavage by Drosha is more important because Dicer just cleaves double-stranded RNA (dsRNA) at the site of ~22 nt from the cleavage sites of Drosha [20]. The big terminal loop and the stable double-strand of an RNA hairpin are two main requirements for Drosha to effectively cleave pri-miRNA, and an SNP which destroys the two requirements is supposed to block miRNA biogenesis.

Case–control study is a main route for searching disease-related SNPs. By case–control study, some SNPs in genes associated with schizophrenia are revealed [21], some SNPs in pre-miRNA (miRNA precursor) flanking regions are discovered to be associated with schizophrenia or breast cancer [6,10], and some SNPs in pre-miRNAs or in miRNA seeds are detected to cause coronary artery disease [9]. In contrast to traditional case–control studies which specifically test one or a few genetic regions, GWAS investigates the entire genome. For example, Jing Gong et al. performed a genome-wide scan for SNPs in human pre-miRNAs, miRNA flanking regions, and target mRNAs, and designed a pipeline to predict the effects of them on miRNA/mRNA interaction [22]; Keren Oved et al. identified CHL1 as a tentative selective serotonin reuptake inhibitors by genome-wide expression profiling of human lymphoblastoid cell lines (LCLs) [23]; and Jesse D. Ziebarth et al. constructed a database, PolymiRTS Database 2.0, which provided links between SNPs in miRNA target sites and the results of GWAS of human diseases [24]. The studies above have discovered disease-related SNPs in miRNAs, but the complicated steps including sample collection, biochemical assay and long-period observation become a limit of convenient and fast detection of disease-related SNPs in miRNAs. Moreover, the studies generally detect the SNPs whose rare frequency is more than 1%, so the rarer SNPs cannot be discovered.

Among a multitude of complex diseases, schizophrenia might be closely associated with miRNA mutation. Different from many complex diseases with obvious changed expression of a few genes, schizophrenia follows many genes with small expression alteration [10]. Because no core genes with significant expression alternation contribute to a big risk of schizophrenia, we might hypothesize that dysregulation of genes could play a more important role in schizophrenia than altered expression genes themselves [10]. As an important regulator of genes, a single miRNA can regulate expression of multiple genes, an SNP in schizophrenia-associated miRNAs (SZmiRNA) can therefore change the expression of multiple genes even if the expression change of each gene is not significant. According to it, we believe that an SNP in SZmiRNAs has a high possibility to cause schizophrenia. Moreover, schizophrenia is a severe disabling brain disease, and miRNAs have been proved to be involved in brain development and function [10,25], therefore, SNPs in SZmiRNAs, a main mutation of miRNAs, are also supposed to be closely associated with schizophrenia. However, the association of SNPs in miRNAs and schizophrenia is unexplored due to poor understanding of the pathophysiology and molecular mechanisms of schizophrenia even if there have been some recent researches focusing on the identification and analysis of SNPs in miRNAs [15,22,26-28].

Identification of schizophrenia-causal SNPs (SZ-SNP) is necessary for further study of schizophrenia. In this study, we propose a novel and simple computational method to detect SZ-SNPs in SZmiRNAs by detecting free energy change caused by the existence of an SNP. Using the method, the SNPs which can change free energy significantly are discovered, and 6 out of 32 known SNPs in SZmiRNAs are supposed to be responsible for most cases of schizophrenia in population.

## Results

### Identified SNPs associated with schizophrenia

We collect 20 SZmiRNAs, and add an SNP into them by replacing every nucleotide with any one of the other three letters after dividing each SZmiRNA into 3 segments: terminal loop, mature miRNA duplex and extension duplex (see Methods). There are separately 1284, 3585, and 4065 SNPs in the three segments of the SZmiRNAs. By detecting free energy change of each segment, 176 (13.7%) SNPs in terminal loops can decrease free energy, and 2310 (64.4%) SNPs in mature miRNA duplexes and 1775 (43.7%) SNPs in extension duplexes can increase free energy. Although the 4261 (176 + 2310 + 1775) SNPs might be associated with schizophrenia more or less, many of them are not causal ones because some just change free energy very slightly. We sort the SNPs in terminal loops by the free energy change, and then compute (sum of free energy change of top n (n = 1,2, …, 176) SNPs/sum of all free energy change) to find the inflection point of free energy change. The top 57 SNPs in terminal loops are supposed to be causal ones. Similarly, the top 533 SNPs in extension duplexes and 888 SNPs in mature miRNAs are identifies as SZ-SNPs. From the SZ-SNPs (see Additional file 1), we find that free energy is significantly decreased when nucleotides A and U are changed and free energy is significantly increased when nucleotides C and G are changed. This phenomenon comes from that there are three hydrogen bonds between a C-G base pair and two bonds between an A-U base pair, therefore, forming a new or destroying an available C-G pair can cause larger change in free energy than an A-U pair.

### Identification of SNPs responsible for most cases of schizophrenia

There are two groups of SNPs in our study: known SNPs which are those detected in former studies, and artificial SNPs which are never reported. By analyzing free energy change caused by SNPs (see Table 1), we find there seems no significant difference between the known and artificial SZ-SNPs. The range of free energy increase caused by all the SZ-SNPs in mature miRNAs is from to 1.5 to 4.7 (average free energy increase is 6.0), and the range of free energy increase caused by the known SZ-SNPs is from 1.7 to 3.4 (average free energy increase is 2.5). The range of free energy increase caused by all the SNPs in extension duplexes is from to 2.7 to 18 (average free energy increase is 6.0), and the range of free energy increase caused by the known SZ-SNPs is from 2.7 to 18 (average free energy increase is 8.4). We perform a *t*-test and prove that free energy change caused by the known SZ-SNPs is not significantly different from that caused by the artificial SZ-SNPs, but a known SZ-SNPs have higher possibility to be causal for most cases of schizophrenia than an artificial one even if free energy change caused by them is same. People could have schizophrenia if they has any SZ-SNP, either known or artificial, but there could be few people if a SZ-SNP has very low minor allele frequency, therefore, the frequency of a SNP has to be taken into account for identifying causal SNPs in most cases of schizophrenia in population. We think artificial SNPs have a lower minor allele frequency than known SNPs because they never been detected.

**Table 1:** Free energy change caused by SNPs

**Segment**	**SNP**			**SNP with changed free energy**			**SZ-SNP**		
	**Number**	**Range of free energy change**	**Average free energy change**	**Number**	**Range of free energy change**	**Average free energy change**	**Number**	**Range of free energy change**	**Average free energy change**
Terminal loop	1284	[-6.2, 5.5]	0.08	176	[-6.2, 0)	-1.1	57	[-6.2,-1.3]	-2.5
Mature miRNA	3585	[4.7, -3.4]	0.56	2310	[4.7, 0)	1.3	888	[4.7, 1.5]	2.2
Extension duplex	4065	[18.2, -10.9]	0.6	1775	[18.2, 0)	2.6	533	[18.2,2.7]	6.0

SNPs with changed free energy are those causing decrease in free energy of terminal loops and those causing increase in free energy of mature miRNAs and extension duplexes.

According to the minor allele frequency of SNPs, we divide them into three categories: known SNPs with known frequency, known SNPs with unknown frequency, and artificial SNPs. We firstly estimate a random frequency in the known frequency range of the known SNPs for the known SNPs with unknown frequency, and estimate a random frequency between 0 and the smallest known frequency of the known SNPs for the artificial SNPs. We then compute (frequency*free energy change) as a score for each SNP, and sort the SNPs by the score. The process of frequency estimation, score computation and SNP sorting is operated 1000 times, the top SNPs accounting for 50% of sum of scores (sum of negative scores in terminal loops and sum of positive scores in extension duplexes and mature miRNAs) with p-value < 0.05 are supposed to be SZ-SNPs responsible for most cases. Among the SZ-SNPs, there are 6 SNPs are discovered, and all of them are known (see Table 2).

**Table 2:** SZ-SNPs responsible for most cases of schizophrenia

**miRNA**	**Strand**	**Segment**	**Sequence**	**SNP ID**	**Allele**
hsa-miR-198-5p	-	mature miRNA	GGUCCAGAG ***G*** GGAGAUAGG	rs142303836	G/A
hsa-miR-92b-3p	+	mature miRNA	UAUUGCACUCGUCCCG ***G*** CCUCC	rs12759620	G/C
hsa-miR-182	-	extension duplex	UGGGGCGAGGACUCAGCC ***G*** GCACCC	rs76481776	G/A
hsa-miR-182	-	extension duplex	UGGGGC ***G*** AGGACUCAGCCGGCACCC	rs374455999	G/A
hsa-miR-182	-	extension duplex	GGGGAGCUGCUUGCCUCC ***C*** CCCGUU	rs370756213	C/U
hsa-let-7 g	-	extension duplex	CAGGAACAGCGCGCCA ***G*** CUGCCAAG	rs9631505	G/A

SNPs in sequences are highlighted in bold and italic.

One SZ-SNP (rs76481776) among six SZ-SNPs responsible for most cases of schizophrenia has been detected to be associated with major depression and schizophrenia in reference [29], and three known SZ-SNPs (rs80041074, rs77586312, and rs75953509) were discovered in patient samples [30].

## Discussion

### SZmiRNAs without significant or known SZ-SNPs

Although all 27 SZmiRNAs are reported to be associated with schizophrenia [10,29-35], SNPs in them do not have same close association with schizophrenia.

First, some SZmiRNAs do not contain significant SZ-SNPs. There are separately 19, 27, and 19 unique SZmiRNAs which contain significant SZ-SNPs in terminal loops, mature miRNA and extension duplexes. For the SZmiRNAs which do not contain significant SZ-SNPs, SNPs in miRNAs might not be the main ways to be associated with schizophrenia. Some SZmiRNAs contain more significant SZ-SNPs than others, so the SNPs in the miRNAs are more likely to have a stronger association with schizophrenia. Second, there are four SZmiRNAs (*hsa-miR-198-5p*, *hsa-miR-92b-3p*, *hsa-miR-182*, and *hsa-let-7 g* in Table 2) could be largely responsible for most cases of schizophrenia caused by SNPs in miRNAs.

As for the SZmiRNAs which contain no SZ-SNPs in some segments, they are still associated with schizophrenia. First, SNPs in other regions, such as schizophrenia-associated genes (SZGenes) or 1 k flanking regions of SZmiRNAs, can cause abnormal expression of SZGenes or SZmiRNAs, thus are associated with schizophrenia. Second, other mutation, such as copy number variation (CNV), or DNA methylation, are also responsible for schizophrenia [36,37].

### High frequency of known SNPs in SZmiRNAs

The total length of 27 SZmiRNAs is 3009 bases, and in which there are 35 known SNPs, thus the frequency of known SNPs in the 27 SZmiRNAs is 1.16% which is much higher than the average estimate that SNPs occur 1 in 1000 base pairs.

Uneven distribution of SNPs in genome might account for the high frequency of SNPs in SZmiRNAs. First, SNPs usually occur more frequently in non-coding regions than in coding regions [2,22] and miRNAs are very important regulators in non-coding regions, so the frequency of SNPs in SZmiRNAs should be higher than in coding regions. Second, compared with genes, miRNAs haven the more profound and broader influence on natural selection which is acting and fixating the allele of the SNP that constitutes the most favorable genetic adaptation [2,22], thus having a higher frequency of SNPs. Moreover, schizophrenia is likely caused by many genes which individually contribute a small risk [25]. A single SNP in SZmiRNA can change expression of many SZGenes by changing the SZmiRNA/SZGene interaction, so SNPs in SZmiRNAs are more possible to cause schizophrenia than SNPs in other regions [25]. High frequency of SNPs in SZmiRNAs can also explain the important role of miRNA in the etiology of schizophrenia.

### Uneven distribution of known SNPs in SZmiRNA segments

The frequencies of the known SNPs in terminal loops, mature miRNA and extension duplexes of SZmiRNAs are 11.68 SNPs/kb (5), 4.18 SNPs/kb (5) and 16.24 SNPs/kb (22) separately, and frequencies of the known SZ-SNPs responsible for most cases of schizophrenia in the 3 segments are 0 SZ-SNPs/kb (0 SNP), 1.67 SZ-SNPs/kb (2) and 2.95 SZ-SNPs/kb (4) separately. The frequencies of known SNPs in the 3 segments of SZmiRNAs are listed in Table 3, from which, we find that the frequency of the known SNPs in extension duplexes is higher than that in terminal loops and mature miRNA duplexes. The higher frequency of known SNPs and SZ-SNPs in extension duplexes suggests that SNPs might be prone to occur in extension duplexes and unstable structure of extension duplexes might have profounder influence on miRNA biogenesis than a small terminal loop.

**Table 3:** known SNP and SZ-SNP frequencies in SZmiRNAs

**Region**	**number of SNPs**	**Mean frequency (number/kb)**	**number of SZ-SNPs**	**Mean frequency (number/kb)**
Terminal loop	5	11.68	0	0
Mature miRNA	5	4.18	2	1.67
Extension duplex	22	16.24	4	2.95

### None of known SNPs which can decrease free energy of terminal loops

None of the five known SNPs in terminal loops can decrease free energy, but we cannot draw the conclusion that the known SNPs in terminal loops of SZmiRNAs are not associated with schizophrenia because the structure of a terminal loop predicated by software is not accurate. Reference [38] indicates that a real miRNA hairpin structure often holds a large (> = 10 nt) and unstructured terminal loop which is a good substrate for efficient cleavage by Drosha, but the size of terminal loop predicted by software is usually small (<=5 nt). For instance, using RNAfold [39] (a software to predict the secondary structure of a single stranded RNA or DNA sequences), *hsa-let-7 g*, *hsa-mir-7-1* and *hsa-mir-9-2* are predicted to have a very small terminal loop with the size of 4 nt, and *hsa-mir-29c* is predicted to have a terminal loop with the size of 5 nt. Therefore, the proposed method based on analysis of free energy for detecting SZ-SNPs does not perform in terminal loops as well as in mature miRNA and extension duplexes.

## Conclusions

Some SNPs in SZmiRNAs can change the internal energy of miRNAs by changing miRNA secondary structure or miRNA/mRNA interaction, which can cause abnormal expression of SZmiRNAs and SZGenes, we therefore think that there is a causal link between SNPs in SZmiRNAs and schizophrenia. Moreover, it is feasible to discover causal SNPs by investigating free energy change because free energy is an indicator of structural stability. The proposed method based on free energy for identifying causal SNPs in SZmiRNAs is not only convenient because biochemical assay or sample comparison between cases and controls are not necessary, but it also has high resolution for causal SNPs even if the SNPs never been reported because they are very rare in the population. In addition, the proposed method can be applied to discover the causal SNPs in miRNAs associated with other diseases.

## Methods

Hairpin is the typical structure of a miRNA, which is also known as stem-loop structure. In the structure, base pairs form a double helix (stem) that ends in an unpaired loop (loop). The unpaired loop at the end is the terminal loop, and two mature miRNAs originated from opposite arms of a same miRNA can inhibit gene expression by binding them with 3′UTR of mRNAs. The stable hairpin of a miRNA and stable base-pairing between mature miRNA and target mRNA are the basis for successful cleavage of mature miRNA and inhibition of target mRNA expression, but the stable structures could be changed by SNPs in the miRNA. If target mRNAs of a miRNA are associated with some diseases, the variation in structure stability of a miRNA might cause diseases because expression of target mRNAs cannot be inhibited normally.

An SNP in a miRNA is supposed to be a causal or functional one if it blocks miRNA maturation or hampers miRNA/mRNA interaction, which often presents in a structural variation of the miRNA. In the hairpin of a miRNA, a large terminal loop and stable double-stranded structure of extension duplex (25 nt upstream and downstream sequences from cleavage sites of mature miRNA duplex) are important for miRNA maturation because a large (> = 10 nt) terminal loop [38,40] and stable double-stranded structure of an extension duplex make a good substrate for efficient cleavage of a miRNA. An SNP in a terminal loop or an extension duplex can block maturation of miRNA, and result in abnormal expression of target genes for no enough mature miRNA for binding target genes if it decreases the size of the terminal loop or impairs stability of the extension duplex. Moreover, an SNP in mature miRNA can cause abnormal expression of target genes if it destroys base pairs between mature miRNA and 3′UTR of target genes. However, a miRNA mutant with changed structure but retained stability still supports cleavage [18] and miRNA/mRNA interaction, therefore, only the SNPs which can impair stability are considered to be causal.

According to the effect of different parts of a miRNA sequence on miRNA biogenesis and miRNA/mRNA interaction, a miRNA is divided into three segments: terminal loop, mature miRNA duplex and extension duplex. Some structure variations of the three segments with changed stability, such as decrease in the size of a terminal loop, damage in stability of an extension duplex, and destruction of base pairs between miRNA and target genes, can be evaluated conveniently with free energy. Free energy is a reflection of structural stability, and it has been widely used in target prediction, function analysis of miRNA and miRNA/mRNA interaction [17,41-43]. In the hairpin of a miRNA, free bases unpaired with any bases are more active than paired bases because they are easier to act with other free bases, thus accounting for structural instability. Generally, the lower free energy a RNA strand possesses, the more stable its structure is. Free energy here can be used to evaluate the stability of the hairpin of a miRNA and miRNA/mRNA interaction. Although structure variation could not accompany a changed free energy, and a structure variation with an unchanged free energy has few effect on stability [18], change in free energy does follow structure variation, and a structure variation with changed free energy must change stability.

An SNP in a SZmiRNA can be identified as a causal one by detecting free energy change from addition of addition of the SNP.

**Figure 2:**
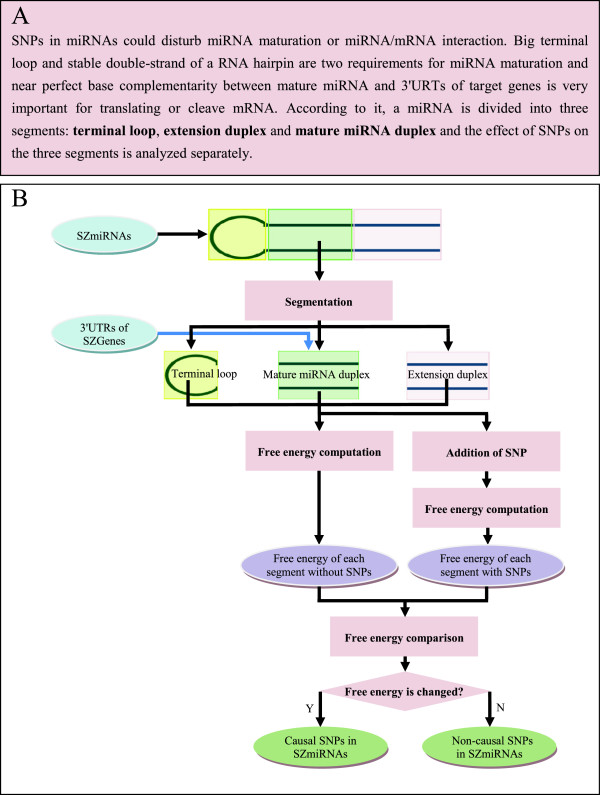
**Flowchart of the method. (A)** Basis of segmentation, **(B)** Flowchart to search causal SNPs. Based on the requirements of miRNA maturation and miRNA/mRNA interaction in **A**, a miRNA is divided into three segments: terminal loop, mature miRNA duplex and extension duplex in **B**. **B** presents the procedure to discover causal SNPs in detail.

The proposed method for SZ-SNPs in SZmiRNAs based on free energy detection is composed of three steps: SZmiRNA segmentation, SNP addition, and free energy change detection.A flowchart of identification procedure is shown in Figure 2.

### Collection of SZmiRNAs, SZGenes and SNPs in SZmiRNAs

Most SZmiRNAs and all SZGenes are downloaded from Schizophrenia Gene Resource (SZGR, http://bioinfo.mc.vanderbilt.edu/SZGR/), hsa-miR-30e and hsa-miR-182 are collected from some studies associated with mental illness [10,29,30,33-35] (see Additional file 2 and Additional file 3). All the SNPs in the SZmiRNAs are gathered from NCBI (the National Center for Biotechnology Information advances science and health by providing access to biomedical and genomic information, http://www.ncbi.nlm.nih.gov) (see Additional file 4).

### Segmentation of SZmiRNAs

**Figure 3:**
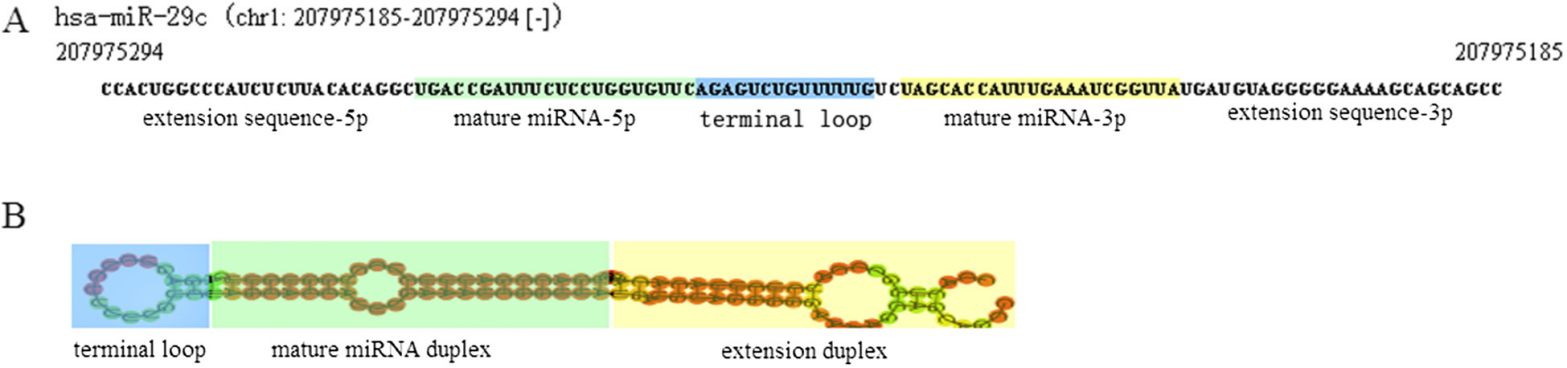
**Segmentation of hsa-miR-29c. (A)** Sequence of hsa-miR-29c, **(B)** Secondary structure of hsa-miR-29c. The sequences in green and in yellow in **A** are mature miRNA-5p and mature miRNA-3p which compose mature miRNA duplex in **B**. The extension sequence-5p and extension sequence-3p in **A** compose extension duplex in **B**.

**Figure 4:**
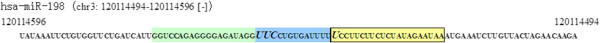
**Segments of hsa-miR-198.** Mature miRNAs annotated artificially are shown in box and overlapping bases are shown in larger font size and italic.

Each SZmiRNA is divided into three segments: terminal loop, mature miRNA duplex, and extension duplex. For example, the segmentation of *hsa-miR-29c* is shown in Figure 3. The segmentation sites of a SZmiRNA are determined based on annotations in miRBase (a miRNA database, http://www.mirbase.org), but there are two exceptional cases. First, some SZmiRNAs have only one mature miRNA sequence annotated in miRBase (e.g. *has-miR-206*, *has-miR-30d*, and *has-miR-7-3*). We must artificially annotate another mature miRNA sequence for them. Two general principles are used for the artificial annotation of the mature miRNA sequence: a 2 nt overhang at the start site of mature miRNA-5p sequence and the end site of mature miRNA-3p, and good complementarity between two mature miRNA sequences. Second, there could be overlap between different segments, for instance, *hsa-miR-198* has an overlap of three bases between mature miRNA-5p and terminal loop. The overlap of two segments should be contained in both two segments. Segmentation of *hsa-miR-198* is shown in Figure 4.

### Detection of free energy change from addition of an SNP

SZ-SNPs are identified by investigating free energy change of each segment caused by the addition of an SNP. After the free energy of each original segment is computed, an SNP is added into each segment by replacing every nucleotide with any one of the other three letters, and the new free energy of the segment with an added SNP is computed. The free energy change caused by the SNP is finally obtained. Below, we analyze the effect of an SNP on the three segments separately.

**Figure 5:**
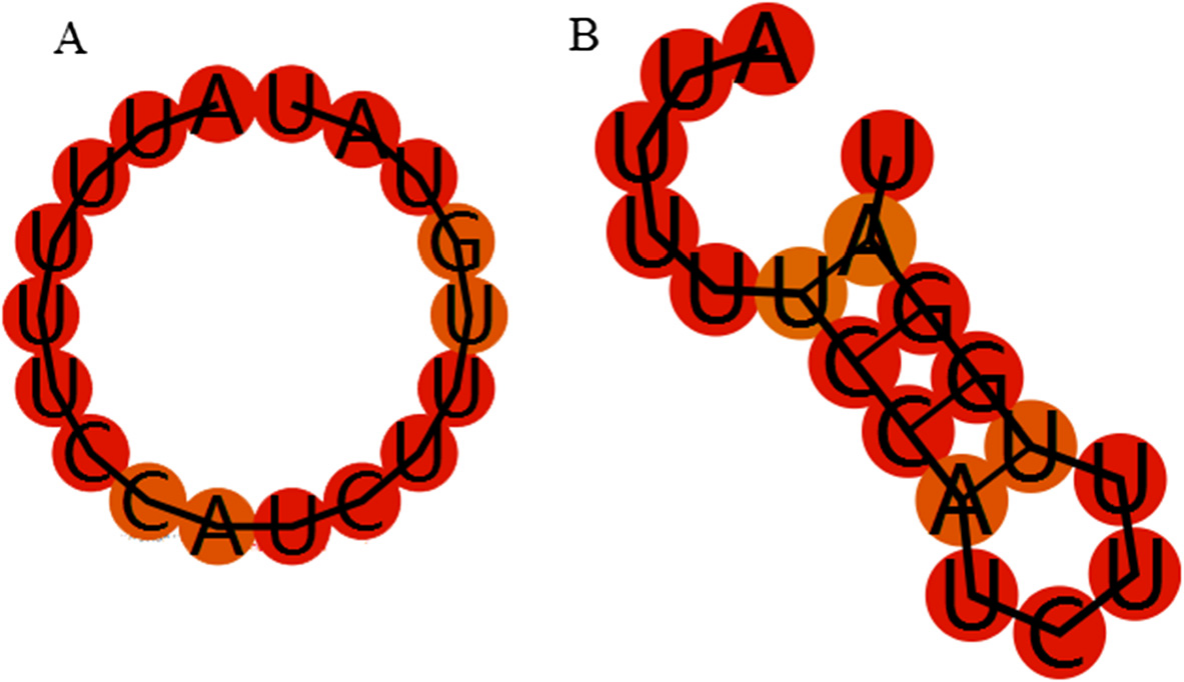
**Secondary structure of terminal loop of hsa-miR-29b-2. (A)** Secondary structure of original terminal loop of hsa-miR-29b-2, **(B)** Secondary structure of terminal loop of hsa-miR-29b-2 with the sixteenth base varied from U to G. Both the two secondary structures are predicted by RNAfold (software to predict the secondary structures of single stranded RNA or DNA sequences).

First, the effect of an SNP on the size of a terminal loop is analyzed. Terminal loop is an unstructured loop in the hairpin of a miRNA, that is, no bases can be paired with others in a terminal loop. An SNP which can decrease the size of a terminal loop is supposed to be a causal one because a large terminal loop is a good substrate for miRNA maturation. If an SNP in a terminal loop makes some bases pair with each other, the size of the terminal loop will be decreased because the complementary bases can form base pairs and they are mistaken for a part of the stem of the hairpin. For example, the sixteen nucleotide variation from U to G in the terminal loop of *hsa-miR-29b-2* (AUUUUUCCAUCUUUGUAU) significantly decreases the size of the terminal loop from 16 to 4 (Figure 5). The decreased size of a terminal loop means there appear fewer free bases, therefore the terminal loop with decreased size certainly follows a decrease in free energy. We firstly compute the free energy of the original terminal loop of a SZmiRNA and that of the terminal loop after adding an SNP, then we can get the free energy change caused by the SNP. The SNPs which can decrease free energy of terminal loops of SZmiRNAs are supposed to be associated with schizophrenia because they can decrease the size of terminal loops and block SZmiRNA maturation. Here, the function rnafold() in Bioinformatics toolbox in Matlab is applied to compute the free energy of terminal loops.

Second, the effect of SNPs in extension duplexes on structural stability can also be evaluated by free energy. Because the stable double-strand of an extension duplex is one of the requirements for miRNA maturation, an SNP in an extension duplex is supposed to be a causal one if it destroys the stability of the extension duplex by destroying base pairs. Destruction of base pairs inevitably releases free bases, and leads to increase in free energy, therefore, the SNPs in extension duplexes which can increase free energy are identified as SZ-SNPs. Here, we also use the function rnafold() to predict free energy of an extension duplex, but the function can only predict free energy of a single strand, and there are two separate sequences in an extension duplex. According to the reference [44], a linker “GGCGGGG” can be inserted between the two sequences of an extension duplex to compose a single strand before computing free energy.

Third, the effect of SNPs on miRNA/mRNA interaction is also evaluated by free energy. Mature miRNAs can inhibit protein translation of target mRNAs by binding themselves to 3′UTR of target mRNAs, and good sequence complementarity between miRNAs and target mRNAs is a basic requirement for binding. An SNP in a mature miRNA could also release free bases by destroying base pairs between the mature miRNA and 3′UTRs of its target mRNAs, and cause increase in free energy. Because 3′UTR of a mRNA is a much longer RNA sequence than miRNA and the function rnafold() cannot find the most complementary short sequence in 3′UTR with mature miRNA, RNAhybrid [45] (a on-line tool for finding the minimum free energy hybridization of a long and a short RNA, http://bibiserv.techfak.uni-bielefeld.de/rnahybrid/submission.html) is applied to compute free energy between a mature miRNA and its target mRNAs.

The detection of a SZ-SNP in a mature SZmiRNA duplex follows the three steps. Step 1, the software Targetscan [46] (http://www.targetscan.org/vert_61, a software to search for predicted miRNA targets in mammals) is used to predict all target genes of a SZmiRNA, from which, the genes also appear in SZGenes are selected as target SZGenes (see Additional file 5). Step 2, all 3′UTRs of the SZGenes are downloaded from UTRdb (a curated database of 5′ and 3′ untranslated sequences of eukaryotic mRNAs, http://utrdb.ba.itb.cnr.it/) and free energy between mature SZmiRNAs and 3′UTRs of target SZGenes is computed by using RNAhybrid. Moreover, one gene might have multiple different 3′UTRs due to multiple transcripts of a gene, but only the 3′UTR with the lowest free energy between which and mature miRNA is taken into account in next step due to the high similarity of 3′UTRs among different transcripts of a gene. Step 3, the average free energy between a mature SZmiRNA and its all target SZGenes is computed, and the SNPs in mature SZmiRNAs which can increase average free energy are supposed to be associated with schizophrenia.

## Competing interests

The authors declare that they have no competing interests.

## Authors’ contributions

XS designed the project, collected and analyzed the data and wrote the manuscript. JZ supervised the study and revised the manuscript. Both authors critically reviewed and approved the final manuscript.

## Supplementary Material

Additional file 1 **Identified SZ-SNPs.** There are identified SZ-SNPs in terminal loops, mature miRNAs and extension duplexes of SZmiRNAs. Click here for file

Additional file 2 **SZmiRNAs.** SZmiRNAs are collected from 8 studies associated with mental illnesses. Click here for file

Additional file 3 **SZGenes.** SZGenes are collected from 4 literatures. Click here for file

Additional file 4 **SNPs in SZmiRNAs.** SNPs in SZmiRNAs are collected from NCBI. Click here for file

Additional file 5 **Regulatory relation between SZmiRNAs and SZGenes.** Target SZGenes are the genes both predicted by the software Targetscan and appeared in SZGenes. Click here for file
