# Supplementary material for: Identification of putative pathogenic SNPs implied in schizophrenia-associated miRNAs
Source: BMC Bioinformatics. 2014 Jun 17;15:194. doi: 10.1186/1471-2105-15-194 (PMC4072616; doi:10.1186/1471-2105-15-194)
Supplement: Additional file 2 — SZmiRNAs. SZmiRNAs are collected from 8 studies associated with mental illnesses. [file 1471-2105-15-194-S2.doc]

| SZmiRNAs | | |
| --- | --- | --- |
| MiRNA Family | MiRNA | |
| hsa-let-7/98/4458/4500 | hsa-let-7g | hsa-let-7g |
| hsa-miR-29abcd | hsa-miR-29a | hsa-miR-29a |
|  | hsa-miR-29b | hsa-miR-29b-1 |
|  |  | hsa-miR-29b-2 |
|  | hsa-miR-29c | hsa-miR-29c |
| hsa-miR-9 | hsa-miR-9 | hsa-miR-9-1 |
|  |  | hsa-miR-9-2 |
|  |  | hsa-miR-9-3 |
| hsa-miR-26ab/1297/4465 | hsa-miR-26b | hsa-miR-26b |
| hsa-miR-30abcdef/30abe-5p/384-5p | hsa-miR-30b | hsa-miR-30b |
|  | hsa-miR-30d | hsa-miR-30d |
|  | hsa-miR-30e | hsa-miR-30e |
| hsa-miR-198 | hsa-miR-198 | hsa-miR-198 |
| hsa-miR-1ab/206/613 | hsa-miR-206 | hsa-miR-206 |
| hsa-miR-25/32/92abc/363/363-3p/367 | hsa-miR-92b | hsa-miR-92b |
| hsa-miR-132/212/212-3p | hsa-miR-212 | hsa-miR-212 |
| hsa-miR-7/7ab | hsa-miR-7 | hsa-miR-7-1 |
|  |  | hsa-miR-7-2 |
|  |  | hsa-miR-7-3 |
| hsa-miR-181abcd/4262 | hsa-miR-181b | hsa-miR-181b-1 |
|  |  | hsa-miR-181b-2 |
| hsa-miR-24 | hsa-miR-24 | hsa-miR-24-1 |
|  |  | hsa-miR-24-2 |
| hsa-miR-15abc/16/16abc/195/322/424/497/1907 | hsa-miR-195 | hsa-miR-195 |
| hsa-miR-17/17-5p/20ab/20b-5p/93/106ab/427/518a-3p/519d | hsa-miR-106b | hsa-miR-106b |
|  | hsa-miR-20b | hsa-miR-20b |
| hsa-miR-182 | hsa-miR-182 | hsa-miR-182 |

Most SZmiRNAs are collected from two independent studies which identified 25 miRNAs differently expressed in brain cortex of schizophrenia patients compared with control samples using microarray [1-2].

hsa-miR-30e is collected from an independent study which suggests that miR-30e is associated with Schizophrenia [3].

hsa-miR-182 is collected from multiple studies related to mental illness [4-8], in which hsa-miR-182 is considered to be associated with schizophrenia and major depression.

# **Reference**

1. Beveridge NJ, Tooney PA, Carroll AP, Gardiner E, Bowden N, Scott RJ, Tran N, Dedova I, Cairns MJ: **Dysregulation of miRNA 181b in the temporal cortex in schizophrenia.** *Hum Mol Genet* 2008, **17:**1156-1168.

2. Perkins DO, Jeffries CD, Jarskog LF, Thomson JM, Woods K, Newman MA, Parker JS, Jin J, Hammond SM: **microRNA expression in the prefrontal cortex of individuals with schizophrenia and schizoaffective disorder.** *Genome Biol* 2007, **8:**R27.

3. Xu Y, Li F, Zhang B, Zhang K, Zhang F, Huang X, Sun N, Ren Y, Sui M, Liu P: **MicroRNAs and target site screening reveals a pre-microRNA-30e variant associated with schizophrenia.** *Schizophr Res* 2010, **119:**219-227.

4. Im HI, Kenny PJ: **MicroRNAs in neuronal function and dysfunction.** *Trends Neurosci* 2012, **35:**325-334.

5. Potkin SG, Macciardi F, Guffanti G, Fallon JH, Wang Q, Turner JA, Lakatos A, Miles MF, Lander A, Vawter MP, Xie X: **Identifying gene regulatory networks in schizophrenia.** *Neuroimage* 2010, **53:**839-847.

6. Hansen T, Olsen L, Lindow M, Jakobsen KD, Ullum H, Jonsson E, Andreassen OA, Djurovic S, Melle I, Agartz I, et al: **Brain expressed microRNAs implicated in schizophrenia etiology.** *PLoS ONE* 2007, **2:**e873.

7. Kohen R, Dobra A, Tracy JH, Haugen E: **Transcriptome profiling of human hippocampus dentate gyrus granule cells in mental illness.** *Transl Psychiatry* 2014, **4:**e366.

8. Saus E, Soria V, Escaramis G, Vivarelli F, Crespo JM, Kagerbauer B, Menchon JM, Urretavizcaya M, Gratacos M, Estivill X: **Genetic variants and abnormal processing of pre-miR-182, a circadian clock modulator, in major depression patients with late insomnia.** *Hum Mol Genet* 2010, **19:**4017-4025.
