# Supplementary material for: Identification of putative pathogenic SNPs implied in schizophrenia-associated miRNAs
Source: BMC Bioinformatics. 2014 Jun 17;15:194. doi: 10.1186/1471-2105-15-194 (PMC4072616; doi:10.1186/1471-2105-15-194)
Supplement: Additional file 3 — SZGenes. SZGenes are collected from 4 literatures. [file 1471-2105-15-194-S3.doc]

| **SZGenes** | | | | | | | |
| --- | --- | --- | --- | --- | --- | --- | --- |
| **Gene ID** | **Symbol** | **Gene ID** | **Symbol** | **Gene ID** | **Symbol** | **Gene ID** | **Symbol** |
| 23305 | ACSL6 | 148 | ADRA1A | 134265 | AFAP1L1 | 4332 | MNDA |
| 54806 | AHI1 | 207 | AKT1 | 348 | APOE | 4524 | MTHFR |
| 367 | AR | 9826 | ARHGEF11 | 405 | ARNT | 63915 | MUTED |
| 421 | ARVCF | 63827 | BCAN | 627 | BDNF | 10763 | NES |
| 753 | C18orf1 | 10712 | C1orf2 | 718 | C3 | 4762 | NEUROG1 |
| 721 | C4B | 886 | CCKAR | 56990 | CDC42SE2 | 4842 | NOS1 |
| 629 | CFB | 1103 | CHAT | 1113 | CHGA | 9722 | NOS1AP |
| 1114 | CHGB | 1116 | CHI3L1 | 10752 | CHL1 | 4853 | NOTCH2 |
| 89832 | CHRFAM7A | 1139 | CHRNA7 | 1141 | CHRNB2 | 4855 | NOTCH4 |
| 7122 | CLDN5 | 9685 | CLINT1 | 1268 | CNR1 | 64067 | NPAS3 |
| 1312 | COMT | 10814 | CPLX2 | 130749 | CPO | 4867 | NPHP1 |
| 1401 | CRP | 1438 | CSF2RA | 1439 | CSF2RB | 4852 | NPY |
| 1544 | CYP1A2 | 1610 | DAO | 267012 | DAOA | 4835 | NQO2 |
| 2532 | DARC | 1622 | DBI | 780 | DDR1 | 4929 | NR4A2 |
| 1674 | DES | 9993 | DGCR2 | 8214 | DGCR6 | 3084 | NRG1 |
| 27185 | DISC1 | 27121 | DKK4 | 1808 | DPYSL2 | 9542 | NRG2 |
| 1812 | DRD1 | 1813 | DRD2 | 1814 | DRD3 | 4900 | NRGN |
| 1815 | DRD4 | 1816 | DRD5 | 84062 | DTNBP1 | 4908 | NTF3 |
| 1950 | EGF | 1960 | EGR3 | 2029 | ENSA | 22854 | NTNG1 |
| 2065 | ERBB3 | 2066 | ERBB4 | 2161 | F12 | 9253 | NUMBL |
| 79137 | FAM134A | 355 | FAS | 9638 | FEZ1 | 266553 | OFCC1 |
| 2246 | FGF1 | 2335 | FN1 | 93986 | FOXP2 | 10215 | OLIG2 |
| 486 | FXYD2 | 53826 | FXYD6 | 7976 | FZD3 | 4978 | OPCML |
| 2550 | GABBR1 | 2554 | GABRA1 | 2559 | GABRA6 | 5066 | PAM |
| 2561 | GABRB2 | 2566 | GABRG2 | 2568 | GABRP | 5108 | PCM1 |
| 2571 | GAD1 | 2638 | GC | 2730 | GCLM | 5142 | PDE4B |
| 2703 | GJA8 | 54584 | GNB1L | 2890 | GRIA1 | 10611 | PDLIM5 |
| 2893 | GRIA4 | 2894 | GRID1 | 2899 | GRIK3 | 9463 | PICK1 |
| 2900 | GRIK4 | 2902 | GRIN1 | 2904 | GRIN2B | 5289 | PIK3C3 |
| 2913 | GRM3 | 2917 | GRM7 | 2932 | GSK3B | 5305 | PIP4K2A |
| 2936 | GSR | 2937 | GSS | 2944 | GSTM1 | 5321 | PLA2G4A |
| 3094 | HINT1 | 3240 | HP | 55806 | HR | 8398 | PLA2G6 |
| 3269 | HRH1 | 3350 | HTR1A | 3356 | HTR2A | 5362 | PLXNA2 |
| 3359 | HTR3A | 3360 | HTR4 | 3361 | HTR5A | 5521 | PPP2R2B |
| 3586 | IL10 | 3593 | IL12B | 3606 | IL18 | 5533 | PPP3CC |
| 8809 | IL18R1 | 8807 | IL18RAP | 3552 | IL1A | 5624 | PROC |
| 3553 | IL1B | 3557 | IL1RN | 3562 | IL3 | 5625 | PRODH |
| 3565 | IL4 | 3577 | IL8RA | 3623 | INHA | 5803 | PTPRZ1 |
| 51141 | INSIG2 | 3843 | IPO5 | 3702 | ITK | 5902 | RANBP1 |
| 3720 | JARID2 | 3782 | KCNN3 | 8564 | KMO | 51735 | RAPGEF6 |
| 339855 | KY | 51520 | LARS | 4023 | LPL | 5649 | RELN |
| 4049 | LTA | 11178 | LZTS1 | 9223 | MAGI1 | 5999 | RGS4 |
| 9863 | MAGI2 | 260425 | MAGI3 | 7851 | MALL | 23209 | MLC1 |
| 4128 | MAOA | 4133 | MAP2 | 8685 | MARCO | 4277 | MICB |
| 2847 | MCHR1 | 9968 | MED12 | 9443 | MED7 | 84466 | MEGF10 |
| 23322 | RPGRIP1L | 6570 | SLC18A1 | 6532 | SLC6A4 | 8128 | ST8SIA2 |
| 57142 | RTN4 | 6571 | SLC18A2 | 6586 | SLIT3 | 6804 | STX1A |
| 65078 | RTN4R | 6506 | SLC1A2 | 9342 | SNAP29 | 6854 | SYN2 |
| 6271 | S100A1 | 6531 | SLC6A3 | 6663 | SOX10 | 9145 | SYNGR1 |
| 6281 | S100A10 | 7157 | TP53 | 63826 | SRR | 23208 | SYT11 |
| 7857 | SCG2 | 7166 | TPH1 | 7533 | YWHAH | 319100 | TAAR6 |
| 8991 | SELENBP1 | 27037 | TRMT2A | 29801 | ZDHHC8 | 6899 | TBX1 |
| 126669 | SHE | 7257 | TSNAX | 91752 | ZNF804A | 7054 | TH |
| 7353 | UFD1L | 150465 | TTL | 7494 | XBP1 | 7124 | TNF |
| 127933 | UHMK1 | 10628 | TXNIP |  |  |  |  |

The 218 unique genes are collected from 4 literatures (160 by gene ranking using optimal weight matrix, 75 by combined odd ratio method, 38 by manual collection and 173 by gene ranking) [1-4].

**Reference**

1. Sun J, Jia P, Fanous AH, Webb BT, van den Oord EJ, Chen X, Bukszar J, Kendler KS, Zhao Z: **A multi-dimensional evidence-based candidate gene prioritization approach for complex diseases-schizophrenia as a case.** *Bioinformatics* 2009, **25:**2595-6602.

2. Ng MY, Levinson DF, Faraone SV, Suarez BK, DeLisi LE, Arinami T, Riley B, Paunio T, Pulver AE, Irmansyah, et al: **Meta-analysis of 32 genome-wide linkage studies of schizophrenia.** *Mol Psychiatry* 2009, **14:**774-785.

3. Lewis CM, Levinson DF, Wise LH, DeLisi LE, Straub RE, Hovatta I, Williams NM, Schwab SG, Pulver AE, Faraone SV, et al: **Genome scan meta-analysis of schizophrenia and bipolar disorder, part II: Schizophrenia.** *Am J Hum Genet* 2003, **73:**34-48.

4. Sun J, Kuo PH, Riley BP, Kendler KS, Zhao Z: **Candidate genes for schizophrenia: a survey of association studies and gene ranking.** *Am J Med Genet B Neuropsychiatr Genet* 2008, **147B:**1173-1181.
