# Supplementary material for: Identification of putative pathogenic SNPs implied in schizophrenia-associated miRNAs
Source: BMC Bioinformatics. 2014 Jun 17;15:194. doi: 10.1186/1471-2105-15-194 (PMC4072616; doi:10.1186/1471-2105-15-194)
Supplement: Additional file 4 — SNPs in SZmiRNAs. SNPs in SZmiRNAs are collected from NCBI. [file 1471-2105-15-194-S4.doc]

| SNPs in SZmiRNAs | | | | | | | |
| --- | --- | --- | --- | --- | --- | --- | --- |
| SZmiRNA | Strand | SNP ID | Allele | Minor Allele Frequency | Submitted Date | Assay Method | Position |
| hsa-miR-92b | + | rs12759620 | C/G | N.D. | Mar 18, 2004 | WGSA-200403 | chr1:155165044 |
| hsa-miR-9-1 | - |  |  |  |  |  |  |
| hsa-miR-181b-1 | - | rs113162007 | C/G | 0.004 | Oct 16, 2009 | MIRNA-SEQUENCING | chr1:198827998 |
| hsa-miR-29c | - |  |  |  |  |  |  |
| hsa-miR-29b-2 | - | rs200396959 | C/T | 0.001 | Mar 06, 2012 | AGILENTWHOLEEXOME | chr1:207975842 |
| hsa-miR-26b | + | rs79126749 | C/T | 0.025 | Jul 20, 2011 | 1000 Genomes Phase 1 | chr2:219267356 |
| hsa-miR-198 | - | rs142303836 | C/T | N.D. | Jul 20, 2011 | 1000 Genomes Phase 1 | chr3:120114558 |
| hsa-let-7g | - | rs9631505 | C/T | N.D. | Oct 23, 2003 | CHR22_NA17119 | chr3:52302279 |
|  |  | rs145909524 | A/G | N.D. | Jul 20, 2011 | 1000 Genomes Phase 1 | chr3:52302286 |
| hsa-miR-9-2 | - | rs41265488 | A/T | N.D. | Mar 29, 2006 | SI_EXOSEQ_1 | chr5:87962747 |
| hsa-miR-206 | + |  |  |  |  |  |  |
| hsa-miR-106b | - | rs72631827 | G/T | 0.012 | Feb 20, 2013 | EXOME CAPTURE AND SEQUENCING | chr7:99691652 |
| hsa-miR-29a | - |  |  |  |  |  |  |
| hsa-miR-29b-1 | - | rs112938204 | -/C | / | Feb 16, 2010 | / | chr7:130562302 |
|  |  | rs188402254 | A/G | N.D. | Jul 20, 2011 | 1000 Genomes Phase 1 | chr7:130562308 |
|  |  | rs116155675* | C/T | 0.013 | Jul 20, 2011 | 1000 Genomes Phase 1 | chr7:130562314 |
| hsa-miR-30b | - | rs111424617 | C/T | 0.002 | Oct 16, 2009 | MIRNA-SEQUENCING | chr8:135812836 |
| hsa-miR-30d | - | rs142529458 | C/T | N.D. | Jul 20, 2011 | 1000 Genomes Phase 1 | chr8:135817113 |
|  |  | rs66986594 | -/C | / | Mar 06, 2008 | / | chr8:135817116-135817117 |
|  |  | rs185802410 | A/G | N.D. | Jul 20, 2011 | 1000 Genomes Phase 1 | chr8:135817129 |
|  |  | rs188557946 | C/T | N.D. | Jul 20, 2011 | 1000 Genomes Phase 1 | chr8:135817196 |
| hsa-miR-7-1 | - |  |  |  |  |  |  |
| hsa-miR-24-1 | + | rs181496497 | C/T | N.D. | Jul 20, 2011 | 1000 Genomes Phase 1 | chr9:97848343 |
| hsa-miR-181b-2 | + |  |  |  |  |  |  |
| hsa-miR-7-2 | + | rs141312113 | A/G | N.D. | Jul 20, 2011 | 1000 Genomes Phase 1 | chr15:89155064 |
|  |  | rs41276930 | C/T | 0.001 | Mar 29, 2006 | SI_EXOSEQ_1 | chr15:89155073 |
|  |  | rs200523177 | C/G | 0.001 | Mar 06, 2012 | AGILENTWHOLEEXOME | chr15:89155082 |
|  |  | rs147579757 | A/G | N.D. | Feb 20, 2013 | EXOME CAPTURE AND SEQUENCING | chr15:89155121 |
|  |  | rs186890720 | C/T | N.D. | Jul 20, 2011 | 1000 Genomes Phase 1 | chr15:89155162 |
| hsa-miR-9-3 | + |  |  |  |  |  |  |
| hsa-miR-212 | - |  |  |  |  |  |  |
| hsa-miR-195 | - | rs199582948 | C/T | 0.002 | Mar 06, 2012 | AGILENTWHOLEEXOME | chr17:6920922 |
| hsa-miR-7-3 | + | rs146575140 | C/T | N.D. | Feb 20, 2013 | EXOME CAPTURE AND SEQUENCING | chr19:4770747 |
|  |  | rs143035456 | -/GGGGG | / | Mar 11, 2011 | / | chr19:4770695-4770696 |
| hsa-miR-24-2 | - |  |  |  |  |  |  |
| hsa-miR-20b | - |  |  |  |  |  |  |
| hsa-miR-30e | + | rs112439044 | C/T | 0.018 | Oct 16, 2009 | MIRNA-SEQUENCING | chr 1: 40754405 |
| hsa-miR-30e | + | rs373732123 | C/T | N.D. | Feb 20, 2013 | EXOME CAPTURE AND SEQUENCING | chr 1: 40754417 |
| hsa-miR-30e | + | rs370518245 | G/A | N.D. | Feb 20, 2013 | EXOME CAPTURE AND SEQUENCING | chr 1: 40754435 |
| hsa-miR-182 | - | rs76481776* | G/A | 0.063 | Mar 20, 2009 | SEQUENCING PROTOCOL | Chr 7: 129770387 |
| hsa-miR-182 | - | rs77586312 | C/T | 0.001 | Mar 29, 2010 | TRIO_CONSENSUS_CALLS | Chr 7: 129770388 |
| hsa-miR-182 | - | rs374455999 | A/G | N.D. | Feb 20, 2013 | EXOME CAPTURE AND SEQUENCING | Chr 7: 129770400 |
| hsa-miR-182 | - | rs80041074* | G/A | 0.01 | Mar 20, 2009 | SEQUENCING PROTOCOL | Chr 7: 129770399 |
| hsa-miR-182 | - | rs75953509 | C/T | 0.001 | Mar 20, 2009 | SEQUENCING PROTOCOL | Chr 7: 129770395 |
| hsa-miR-182 | - | rs370756213 | C/T | N.D. | Feb 20, 2013 | EXOME CAPTURE AND SEQUENCING | Chr 7: 129770477 |

The starting and ending locations of each SZmiRNA in a chromosome is firstly extracted from NCBI, according to which the SNPs appeared in the SZmiRNA are then searched in dbSNP of NCBI. The three SNPs (rs116155675, rs76481776 and rs80041074) followed by an asterisk have multiple different submission. The minor allele frequency of the three SNPs is the average frequency of different submission, and the submitted date and the assay method of the SNPs is that of the earliest submission.

The SNPs are categorized into two types: single nucleotide variations (SNV) and deletion/insertion variations (DIV). The SNPs whose first character is "-" in allele column are DIVs, others are SNVs. Among the 35 SNPs in the table above, only the 32 SNVs are studied because SNV is a more common case than DIV. SNPs mentioned in this study actually are SNVs.
