# Supplementary material for: Identification of putative pathogenic SNPs implied in schizophrenia-associated miRNAs
Source: BMC Bioinformatics. 2014 Jun 17;15:194. doi: 10.1186/1471-2105-15-194 (PMC4072616; doi:10.1186/1471-2105-15-194)
Supplement: Additional file 5 — Regulatory relation between SZmiRNAs and SZGenes. Target SZGenes are the genes both predicted by the software Targetscan and appeared in SZGenes. [file 1471-2105-15-194-S5.doc]

| **Regulatory relation between SZmiRNAs and SZGenes** | | | | | | | | |
| --- | --- | --- | --- | --- | --- | --- | --- | --- |
| **SZmiRNA** | **Target SZGene** | | | | | | | |
| hsa-let-7g | FOXP2 | ACSL6 | C18orf1 | EGR3 | FAS | FZD3 | TP53 | NOS1 |
|  | NUMBL | GABRA6 | HTR4 | IL10 | MUTED | TTL | SYT11 | KY |
| hsa-miR-29 | NPAS3 | ARNT | ARVCF | C18orf1 | CNR1 | DPYSL2 | SLC1A2 |  |
|  | DRD1 | GSK3B | JARID2 | STX1A | LPL | NOTCH2 |  |  |
| hsa-miR-9 | NOTCH2 | DRD2 | EGR3 | FOXP2 | GABRB2 | SLC18A2 | TSNAX | GAD1 |
|  | PLXNA2 | GRIK3 | GSK3B | KCNN3 | MAGI1 | MEGF10 | STX1A | GCLM |
|  | UHMK1 | NTNG1 | OPCML |  |  |  |  |  |
| hsa-miR-26 | RGS4 | CNR1 | FOXP2 | GSK3B | GSR | JARID2 | PLXNA2 | NRG2 |
|  | KCNN3 | KY | MAP2 | PDE4B |  |  |  |  |
| hsa-miR-30 | RTN4R | BDNF | CHL1 | COMT | DPYSL2 | EGR3 | FZD3 | GRM3 |
|  | MAGI2 | PLXNA2 | SLC6A3 | INSIG2 | JARID2 | KCNN3 | SYN2 | IL1A |
|  | SLC1A2 | MAGI3 | NR4A2 | NRG2 | NTNG1 | PIP4K2A |  |  |
| hsa-miR-198 | SCG2 | CNR1 | DPYSL2 | FZD3 | GRIA4 | PCM1 | SLC6A4 |  |
| hsa-miR-206 | MAGI2 | BDNF | CPLX2 | FAM134A | FN1 | GSK3B | NPAS3 | MCHR1 |
|  | JARID2 | NR4A2 | UHMK1 |  |  |  |  |  |
| hsa-miR-92 | GRIA1 | C18orf1 | CHGA | CNR1 | CPLX2 | FOXP2 | SYN2 | KCNN3 |
|  | GCLM | GRIA4 | JARID2 |  |  |  |  |  |
| hsa-miR-212 | NR4A2 | ARHGEF11 | BCAN | FOXP2 | GRM3 | GSK3B | SLC1A2 | PAM |
|  | KCNN3 | SLC6A3 | NRG2 | NTNG1 |  |  |  |  |
| hsa-miR-7 | FAM134A | C18orf1 | CPLX2 | DPYSL2 | EGR3 | ERBB4 | PDE4B | GABRA1 |
|  | UHMK1 |  |  |  |  |  |  |  |
| hsa-miR-181 | MEGF10 | CNR1 | DGCR2 | DPYSL2 | EGR3 | GABRA1 | PAM | TNF |
|  | GRID1 | GRIK3 | GRM7 | IL1A | IPO5 | JARID2 | SHE | KCNN3 |
|  | NOTCH2 |  |  |  |  |  |  |  |
| hsa-miR-24 | ACSL6 | NOS1 | CHI3L1 | ERBB3 | GAD1 | GRIK3 | MTHFR | MAGI1 |
|  | GSK3B | GRIN1 | HRH1 | IL1A |  |  |  |  |
| hsa-miR-195 | SLC6A4 | BDNF | C18orf1 | CDC42SE2 | DRD1 | FAM134A | HTR2A | TXNIP |
|  | FGF1 | FOXP2 | GRIN1 | GRM7 | HR | UHMK1 |  |  |
|  | STX1A | JARID2 | KY | NOS1 | NOTCH2 | NPAS3 | TTL | SLC1A2 |
|  | HTR4 | PAM | PCM1 | PLXNA2 | RELN | RTN4 | YWHAH | NRG1 |
| hsa-miR-20/106 | TXNIP | ARHGEF11 | DPYSL2 | UHMK1 | EGR3 | ERBB3 | PAM | FZD3 |
|  | ST8SIA2 | ZDHHC8 | GABBR1 | NEUROG1 | NPAS3 | NR4A2 | DRD1 | SLC1A2 |
|  | OPCML | FAM134A | PDLIM5 | PIP4K2A | PLA2G6 |  |  |  |

The target SZGenes are the genes both predicted by the software Targetscan (TargetScan predicts biological targets of miRNAs by searching for the presence of conserved 8mer and 7mer sites that match the seed region of each miRNA, http://www.targetscan.org/vert_61/)and appeared in SZGenes.
